# Predicting Wrist Posture during Occupational Tasks Using Inertial Sensors and Convolutional Neural Networks

**DOI:** 10.3390/s23020942

**Published:** 2023-01-13

**Authors:** Calvin Young, Andrew Hamilton-Wright, Michele L. Oliver, Karen D. Gordon

**Affiliations:** 1 School of Engineering, University of Guelph, 50 Stone Road East, Guelph, ON N1G 2W1, Canada; 2 School of Computer Science, University of Guelph, 50 Stone Road East, Guelph, ON N1G 2W1, Canada

**Keywords:** posture estimation, convolutional neural networks, ergonomic assessment, inertial measurement units

## Abstract

Current methods for ergonomic assessment often use video-analysis to estimate wrist postures during occupational tasks. Wearable sensing and machine learning have the potential to automate this tedious task, and in doing so greatly extend the amount of data available to clinicians and researchers. A method of predicting wrist posture from inertial measurement units placed on the wrist and hand via a deep convolutional neural network has been developed. This study has quantified the accuracy and reliability of the postures predicted by this system relative to the gold standard of optoelectronic motion capture. Ten participants performed 3 different simulated occupational tasks on 2 occasions while wearing inertial measurement units on the hand and wrist. Data from the occupational task recordings were used to train a convolutional neural network classifier to estimate wrist posture in flexion/extension, and radial/ulnar deviation. The model was trained and tested in a leave-one-out cross validation format. Agreement between the proposed system and optoelectronic motion capture was 65% with κ = 0.41 in flexion/extension and 60% with κ = 0.48 in radial/ulnar deviation. The proposed system can predict wrist posture in flexion/extension and radial/ulnar deviation with accuracy and reliability congruent with published values for human estimators. This system can estimate wrist posture during occupational tasks in a small fraction of the time it takes a human to perform the same task. This offers opportunity to expand the capabilities of practitioners by eliminating the tedium of manual postural assessment.

## 1. Introduction

Upper extremity work-related musculoskeletal disorders (WMSDs) are widely prevalent and form a large percentage of all WMSDs [1]. WMSDs, such as carpal tunnel syndrome, often develop and resolve slowly [2]. This would seem like an ideal scenario in which to use wearable sensing for occupational risk assessment, as wearables can collect data persistently and can move with the wearer between environments. This provides a distinct advantage over kinematic monitoring with motion capture, including modern vision based markerless motion capture systems, as these systems are limited to a single predefined capture volume. There has been a rapid proliferation of upper extremity mounted wearable devices such as the Fitbit® or Apple Watch®, meaning that there is already broad familiarity with the use of wearable devices. In their recent survey of occupational health and safety professionals, Schall et al. found that an estimated 27.9% or employees are already using wearable devices while at work, however, this use of wearables is limited to personal activity monitoring (e.g., step counting) rather than for ergonomic assessment [3]. This same survey identified cost, and validity, among others, as barriers to the adoption of wearable technology in workplaces [3]. Despite these barriers, there is widespread interest in using wearable devices to monitor ergonomic risk factors during the performance of occupational tasks [3,4,5].

Upper limb injury risk mitigation has traditionally been done using survey based ergonomic assessment tools. These include tools such as the occupational repetitive action of upper the limb index (OCRA) [6], strain index (SI) [7], rapid upper limb assessment (RULA) [8], and rapid entire body assessment (REBA) [9]. While these indices all take different approaches to risk assessment, common to them all is the requirement that a human observer identify awkward postures. The workflow for each of these assessments involves estimating joint posture visually, and classifying the posture within a specific angular range or bin. A recent study by McKinnon et al. quantified the agreement between human raters performing post-hoc video analysis and electrogoniometer measures of wrist posture [10]. They found an agreement of 57% for flexion/extension (FE), and 68% for radial/ulnar deviation (RU) [10].

Manually identifying wrist postures in frame-by-frame video is a tedious task, making it an ideal candidate for automation. Historically, optoelectronic motion capture, and goniometers have been the primary tools used for objective measurement of joint angular kinematics in the laboratory. In the workplace, electrogoniometers are currently the most commonly used wearable devices for ergonomic assessment using recorded kinematics. Goniometers use a node placed on either side of the joint, connected by either a rigid linkage in the case of an electromechanical goniometer, or a soft flexible transducer in the case of an electrogoniometer [11,12]. Goniometers permit accurate measurement of joint angular position during the performance of occupational tasks. There are, however, several factors which severely limit their utility for longer term, continuous monitoring applications. The first problem is that they are challenging for users to reliably don and doff themselves, meaning any long-term deployment would likely require a practitioner to don the devices, or at minimum verify device placement. Goniometers also require the transducer to cross the joint, and may be adversely affected by external impingement, by for instance, the clothing of the wearer compressing the transducer, which can cause the transducers to report unreliable data [13]. Goniometers are also expensive and rather delicate, which is problematic for long-term deployment in occupational settings. These factors make goniometers unsuitable for long-term continuous ergonomic monitoring.

Advances in inertial measurement unit based wearable systems have generated significant opportunities to collect valid quantitative biomechanical data in out-of-lab environments, including the workplace [14]. IMU based systems have a distinct advantage over electrogoniometers in that capture can be performed with a single, relatively inconspicuous node placed on each biomechanical segment of interest, thus pre-empting the need for a delicate transducer crossing the joint. The performance of these systems, however, is heavily dependent on procedural considerations such as the underlying biomechanical model used, post-hoc drift correction, and sensor to segment coordinate system alignment [15,16]. These procedural considerations mean that a trained practitioner would be required to don the devices. The validity of IMU based systems for measuring joint kinematics have improved significantly but the performance for measuring wrist joint angle has been inconsistent, with reported errors ranging between 2.2° and 30° [17]. Furthermore, well validated IMU systems, such as the Xsens MVN, have costs much closer to traditional optoelectronic motion capture systems. This is a stark contrast to the $72.21 (USD) average acceptable per device cost identified by Schall et al. in their survey of occupational health and safety professionals [3].

One alternative has been to use IMUs in conjunction with surface electromyography (EMG) to predict loading. Peppoloni et al. successfully demonstrated such a system, however, the use of EMG further compounds the procedural difficulty of usage in the workplace [18]. While challenges exist in the direct measurement of wrist kinematics with IMUs, one alternative is to assess metrics other than joint angle such as joint angular velocity, which can be directly measured using gyroscopes placed across the joint as demonstrated by Manivasagam and Yang [19]. This approach offers a much more practical approach to extracting data, however, from a practical standpoint it is still very much the domain of research. Moreover, wrist angular velocity has not been integrated into ergonomic assessment to nearly the same extent as posture. The recent review by Stefana et al., highlights the interest in deploying wearable sensors for ergonomic assessment, however, the use of IMUs for quantifying wrist posture remains uncommon [20].

One strategy for extending the utility of low cost IMUs, which mitigates the problems caused by variability in sensor to segment alignment while also recording postural data, is to process IMU output using machine learning. The practice of deriving joint kinematics using machine learning has been predominantly used for gait analysis [21,22,23]. Results have generally been successful, however, the consistent, periodic nature of gait makes it well suited to this approach. Successful techniques have also relied on segmentation and temporal normalization of the data to a uniform portion of the gait cycle [21]. Kinematics of the wrist do not exhibit the same consistency and periodicity, and as such cannot leverage the same strategies for improving accuracy. Estimating binned wrist postures, rather than a continuous kinematic waveform, is a far more tractable problem that retains much of the practical utility, as it fits well within existing frameworks for ergonomic assessment.

The integration of machine learning within ergonomic assessment is a rapidly growing field. In their 2021 review of machine learning applications to WMSD prevention, Chan et al. found that most of their reviewed work was published in the preceding decade, with almost 25% published in the preceding year [24]. This growth suggests great opportunity, however, models can tend towards sacrificing interpretability for performance, which may ultimately hinder adoption. This provides a powerful motivation for using machine learning to estimate fundamental measures used in ergonomic assessment, such as joint posture, rather than attempting to predict some arbitrary aggregate risk score. Our approach leverages machine learning to produce posture estimates, rather than precise measures of joint position, and although measurement resolution is lost, it greatly simplifies the sensor donning procedure, which will facilitate system deployment in out-of-lab scenarios. Continuous identification of wrist postures would permit the study of large cohorts to quantify the exposure to awkward postures and identify medium- and long-term changes in posture. The purpose of this study was to quantify the performance of a deep convolutional neural network posture classifier for estimating wrist posture during simulated occupational tasks using data produced by two IMUs placed on the hand and wrist as input.

## 2. Materials and Methods

### 2.1. Experimental Procedures

Ten healthy, right hand dominant participants (5 male and 5 female, aged 29 ± 6 years) were recruited for this study. This research complied with the tenets of the Declaration of Helsinki and was approved by the Research Ethics Board at the University of Guelph (REB# 16-12-600). Informed consent was obtained from each participant prior to study participation.

Ground truth wrist posture was collected using a VICON 15 camera (2 Vero, 13 Bonita) optoelectronic motion capture system. Six, 14 mm retroreflective markers were placed to mark the second and fifth metacarpophalangeal joints, radial and ulnar styloid processes, and medial and lateral epicondyles of the elbow. Coordinate systems were defined based on ISB recommendations for the upper extremity [25]. For a full description of coordinate system definitions, please see Young et al. [26].

**Figure 1:**
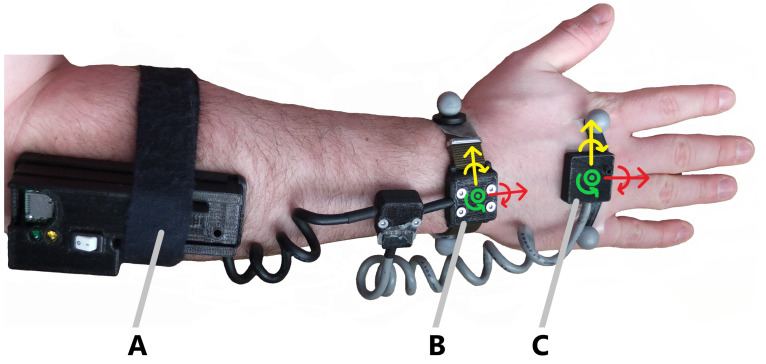
Wearable device showing Datalogger (A), wrist IMU (B), and hand IMU (C). Positive directions for the local sensor coordinate systems are approximately aligned with distal (red) radial (yellow) and dorsal (green) axes.

We have recently developed a low cost (≈100 USD) modular wearable system that consists of a pair of 6 degree of freedom IMUs, and an optional biaxial electromechanical goniometer [26]. The sensors are contained in a 3D printed frame, which can be trivially reconfigured using 3D printing to adapt to either subject or joint specific considerations. In this study the two 6 degree of freedom inertial measurement units were placed on the upper extremity of the dominant arm, one on the dorsal side of the hand with the *x*-axis approximately inline with the proximal-distal axis of the third metacarpal, and one placed on the wrist with the *x*-axis approximately inline with the proximal-distal axis of the forearm (Figure 1). Notably, no specialized sensor-to-segment alignment procedures were performed, other than an approximate visual alignment.

### 2.2. Simulated Occupational Tasks

Participants reported on two occasions and performed two repetitions of three simulated occupational tasks. The first was an assembly/disassembly task, the second was a checkout scanning task, and the third was a typing task. All tasks were performed while standing at a bench with a height of 91.44 cm. Prior to, and immediately following each task, participants were asked to move through their range of motion in wrist FE, RU, and pronation/supination (PS). Task presentation was counterbalanced across participants to mitigate order effects. In this analysis, a total of 120 recordings were analyzed (10 participants × 2 sessions × 2 repetitions × 3 tasks).

#### 2.2.1. Assembly/Disassembly

The assembly/disassembly task consisted of 10 tasks, which could be further broken down into the following 3 task classes: object placement/removal, tightening/loosening fasteners by hand, and tightening/loosening fasteners with a tool. The task order was as follows:

1. Place washers on threaded pegs;
2. Place main body over threaded pegs;
3. Place lateral crossbars over main body;
4. Hand tighten main body retainer nuts onto threaded pegs;
5. Tighten main body retainer nuts with wrench;
6. Place shaft in main body;
7. Place shaft retainer caps over shaft;
8. Tighten shaft retainer cap bolts by hand;
9. Tighten shaft retainer cap bolts with wrench;
10. Disassemble in reverse order.

**Figure 2:**
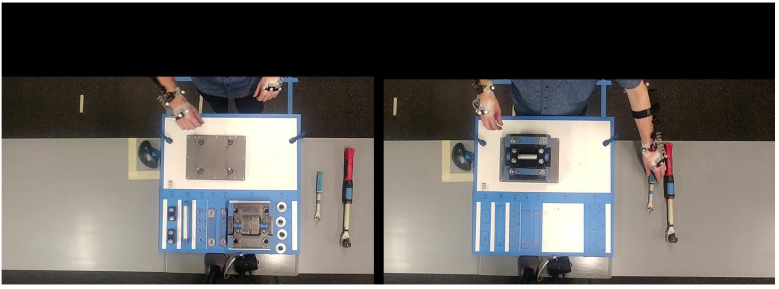
Assembly/disassembly task showing the disassembled apparatus (**left**) and assembled apparatus (**right**).

The experimental apparatus is shown in Figure 2. Participants selected each component from left to right to assemble the widget. Prompts were provided via computer monitor to guide participants through the assembly/disassembly process.

#### 2.2.2. Checkout Scanning

**Figure 3:**
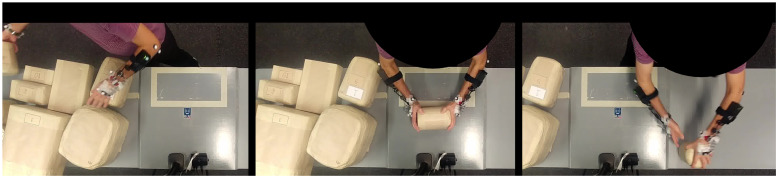
Checkout scanning task showing object selection (**left**), object scanning (**center**), and object placement (**right**).

The checkout scanning task involved selecting and scanning 10 different items of varying size shape and weight. Each item was tagged with a unique radio-frequency identification (RFID) tag, which allowed the item to be scanned. Participants were prompted via a computer screen to select 1 of the 10 items from one end of the workstation. Participants would then touch the RFID tag to the scanner, manipulating the object as necessary, and place the item at the opposite end of the workstation from where it started. The checkout scanning workstation is shown in Figure 3. Participants performed this task twice, ensuring that the object scanning and relocation task took place in both the right-to-left and left-to-right directions.

#### 2.2.3. Typing

**Figure 4:**
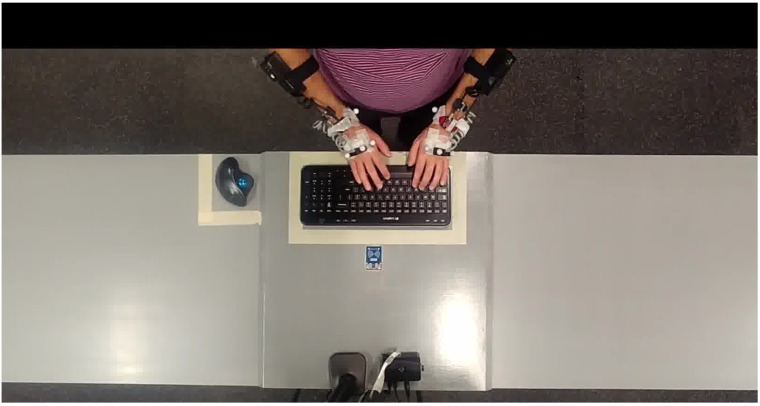
Typing task, showing participant orientation for the 3-minute typing task.

The typing task consisted of three minutes of typing. Participants transcribed text typing at a self selected pace for three minutes while standing at the workstation as shown in Figure 4. The text was drawn from Wikipedia Featured articles.

### 2.3. Data Preprocessing

**Figure 5:**
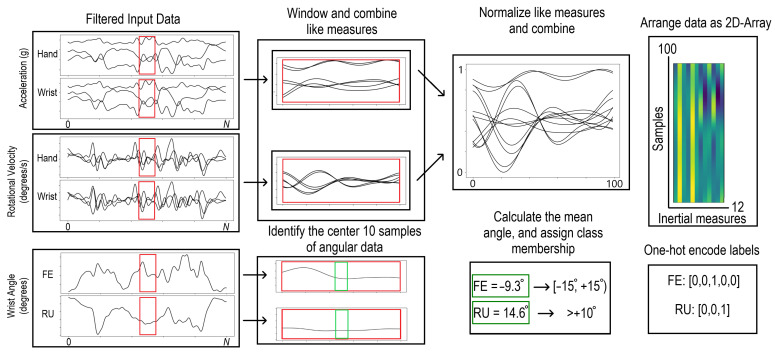
Data preprocessing for network ingestion.

All data from the wearable and from the VICON motion capture system were recorded at 100 Hz. Motion capture derived angular data were filtered using a zero lag Butterworth lowpass filter with a cut-off frequency of 5 Hz, and inertial data were filtered with a zero lag Butterworth lowpass filter with a cut-off frequency of 6 Hz [23]. Acceleration and rotational velocity data were normalized to be between 0 and 1. Inertial data were restructured into 12 × 100 windows consisting of 12 inertial measurands over 100 samples (1 s) with a 90-sample overlap (i.e., 10 windows per second). Detailed preprocessing procedures are shown in Figure 5. The posture associated with each window was computed as the mean of the 10 samples in the center of each window. The mean posture was then binned into one of five postural bins for FE and one of three postural bins for RU. FE bins were θ>45°,45°≥θ>15°,15°≥θ>−15°,−15°≥θ>−45°,−45°≥θ, and RU bins were θ≥10°,10°≥θ>−10°,−10°≥θ. Binned wrist postures are already used within ergonomic assessments such as the SI, RULA, and REBA [7,8,9]. Binning postures in this manner greatly simplifies the estimation problem, while ensuring that this system can produce data which fits within existing ergonomic assessment frameworks. These bins were selected because of their use in previous work, particularly that of Kociolek et al. and Mckinnon et al. in their assessments of the reliability of human raters evaluating wrist postures using video analysis [10,27]. This choice makes it practical to compare directly with the performance of human raters.

After windowing, the dataset contained 183,012 examples, however, the dataset had a significant class imbalance. For FE data, 90% of the data occupied the 15° to −15° and −15° to −45° bins. This extreme class imbalance is unsuitable for neural network training, as the network will tend towards learning the underlying class distribution, rather than the relationship of interest. An additional problem was the variability in the number of examples produced by each participant. This is particularly undesirable as the number of samples contributed by each participant is directly related to the speed at which they performed the tasks. The unbalance could potentially result in the network showing higher performance for lower movement speeds. While it would have been possible to design tasks or provide participants with instructions, which would result in more time spent in extreme postures and a flatter class distribution, it was our preference to have participants interact with the tasks as naturally as possible. It is not unusual that the postures are distributed about an approximately neutral posture, with extreme postures occurring less frequently. This will be a persistent challenge if the goal is to capture a broad range of tasks.

To address the imbalance problems, each task recording was resampled to generate a training set with a more even distribution of class memberships. Each task recording had its angular channel rounded to the nearest integer (i.e., single degree binning). A histogram of the total range of motion used in that recording was then computed, and one example was drawn from each single degree bin. The examples were then randomly sampled with replacement from across the range of motion until a total of 250 examples had been drawn. This resulted in each participant contributing 3000 training examples, which were approximately evenly distributed across the range of motion used.

### 2.4. Neural Network Development

**Figure 6:**
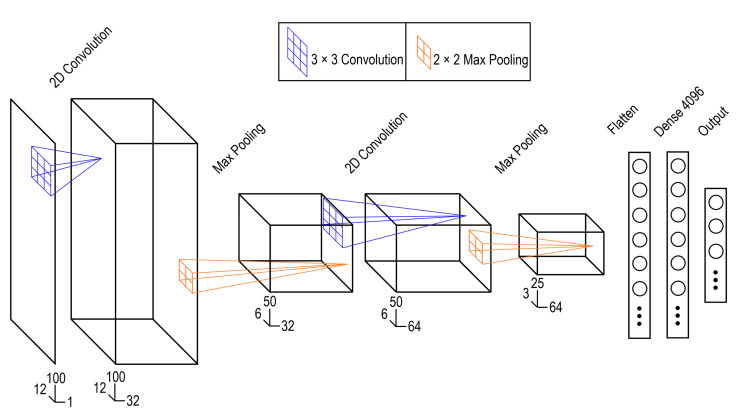
Convolutional neural network architecture showing the convolution (blue) and pooling (orange) operations along with the dimensions of transformed input data.

Data produced by the IMUs were classified into postural bins using a deep convolutional neural network (CNN), the architecture of which is shown in Figure 6. The network was implemented using Keras in TensorFlow 2.8.0. The network used a 32 filter 2d convolutional layer with a 3 × 3 kernel combined with a 2 × 2 max pooling layer, followed by a 64 filter 2d convolutional layer and 2 × 2 max pooling layer. The flattened output of the second max pooling layer was then passed to a fully connected layer with 4096 neurons and a dropout rate of 80%. The network used Rectified Linear Unit (ReLU) activations for the hidden layers with a softmax output activation. The model was run on an Nvidia RTX 2060 Super GPU. Training and evaluation were done in a leave-one-out cross validation format with a refinement step. In this schema the model was retrained 10 times with a different participant used as the test dataset each time. The reported performance is the average performance across all 10 folds.

Initial training was performed with a 10% validation split and we used early stopping to monitor the validation error and stop training if the error failed to improve for 10 epochs. The training had a hard limit of 500 epochs, however, it was completed in fewer than 100 epochs for all 10 folds.

Refinement training was performed on the resampled data drawn from the first visit of the test participant. Refinement also used a 10% validation split; however, the learning rate was 10 times lower, and early stopping was used to stop refinement if the model failed to improve for 1 epoch. Refinement had a hard limit of 50 epochs. Hyperparameter changes between training and refinement were used to avoid overfitting on the refinement data.

The architecture was motivated, in part, by the work done by Mundt et al., which demonstrated success for computing joint kinematics based on inertial measurement units [23]. Mundt et al. opted to use an existing CNN model [23,28]. To make use of this model, it is necessary to format the data as a 224 × 224 × 3 array, which mimics the format of an RGB image. The proposed strategies for this conversion involve upscaling the input data using a cubic spline interpolation [21,23] Our approach, however, uses a far smaller network and does not rely on interpolation to upscale the input data. Instead, the model is trained on the 12 × 100 × 1 array. This simplifies the preprocessing steps, and results in a much smaller input, which is more practical for use in systems with limited memory, such as embedded systems.

## 3. Results

**Figure 7:**
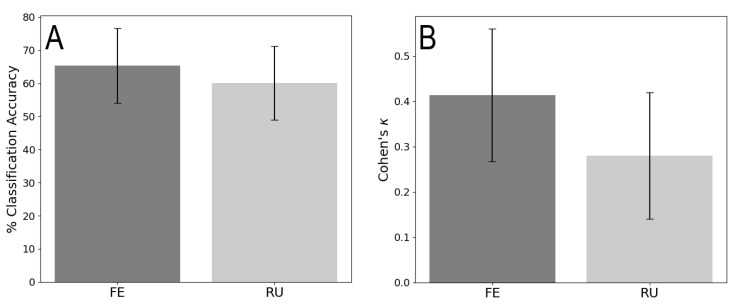
Mean test accuracy (**A**) and Cohen’s κ (**B**) for flexion/extension (FE) and radial/ulnar deviation (RU).

Mean model accuracy and Cohen’s κ with standard error are presented in Figure 7. A precise breakdown of model classification accuracy is shown in Table 1 (FE) and Table 2 (RU).

## 4. Discussion

The performance of the system relative to the ground truth angle was generally favorable. The cumulative performance is similar to that of a human rater, achieving mean accuracies of 65% FE and 60% for RU compared to 57% and 68%, respectively, as reported by Mckinnon et al. [10]. Another important consideration when comparing against human performance is the amount of time required to perform the analysis. On average the recordings produced by each participant contained 18,301 frames, or approximately 30 min of data. The model was able to predict postures from these 18,301 frames in less than 5 s while matching human accuracy. There is in important caveat that the processing time does not include the time required to train the model, however, this still represents a substantial improvement.

Cohen’s κ was computed to assess the reliability of the classifier. The κ statistic is a measure that quantifies the accuracy while accounting for chance agreement due to the underlying distribution of the dataset. Cohen’s κ is predominantly used in the assessment of inter-rater reliability, or classification reliability, and for the latter it is useful for quantifying the performance of a model when assessing unbalanced datasets. Previous work evaluating human performance for posture estimation have adopted the thresholds for κ established by Landis and Koch [10,27,29]. According to Landis and Koch, our mean FE κ score of 0.41 is Moderate, and mean RU κ score of 0.28 is Fair, which is in line with the Moderate and Fair κ scores reported by McKinnon et al. for FE and RU, respectively [10,29]. In general terms κ=0 would indicate random chance agreement, while a κ=1 would indicate perfect agreement. This is useful particularly when studying unbalanced datasets, as a classifier could achieve a high classification accuracy despite consistent erroneous classifications of an improbable class. While the κ score is congruent with the performance of human raters, it also underscores the need to improve and increase the dataset size to more comprehensively represent the postural bins with few observations.

This result is especially compelling given that no special sensor to segment alignment or kinematic calibrations were performed beyond the approximate alignment shown in Figure 1. Typical approaches to deriving accurate kinematics from IMUs involve the use of a sensor-to-segment alignment procedure combined with a mapping of the sensor coordinates to the segment coordinates, either using anatomical measurements or functional movement calibrations, and often use magnetometer data for 9DOF sensors to correct orientation drift [15]. Requiring magnetometer data is problematic in most environments, as variations in local magnetic field will adversely affect the results [16]. Alignment and calibration procedures are also problematic for large scale workplace monitoring studies, as these procedures require specialized training for each participant to be able to don the sensors themselves.

This study used leave-one-out cross validation combined with refinement training. This means that the resultant neural network has been individually matched to the test participant. It is common practice to avoid training on data produced by any participant in the test set if broad generalizability is the goal [30]. There are two primary reasons for this. First, it ensures that the model learns inter-class rather than inter-participant differences, and second, when deployed it is often impractical to collect labeled data to facilitate model refinement for its end user. If collecting data to permit individualized refinement is not possible, then doing so during model evaluation only serves to artificially inflate the model performance. The hardware that we have developed, however, is a unique case, as it is modular, and the electromechanical goniometer module permits the collection of ground truth angular measures [26]. This means that it would be practical to perform an initial occupational assessment using the electromechanical goniometer module, use the collected ground truth data to refine the model for a specific participant, then collect the subsequent angular data using only the inertial measurement units in combination with the CNN for angle prediction.

One challenge associated with this approach is the significant class imbalance in the raw dataset. During the initial training experiments this imbalance resulted in overfitting of the model to the class distribution, rather than the kinematic features of interest. To mitigate this, the class distribution was flattened by resampling the data from each class. Unfortunately, the >45° class accounted for only 0.06% of the total dataset (107 frames) and the very small number of examples likely contributed to the 70% error in that class (Table 1). Unfortunately, no amount of creative resampling can generate more data. This also demonstrates one of the key challenges with this approach, namely, that the most important postures from a risk assessment perspective (i.e., the most extreme postures) also occur the most infrequently. However, it should also be noted that despite the very small number of training examples for the >45° class, 99% of the frames belonging to that class were classified within 1 bin of the true posture. The model also demonstrates excellent performance for the θ≥ −45°, where more data were present (Table 1). This performance discrepancy between the two extreme postural bins suggests that the model could be substantially improved by increasing the number of training examples in the >45° bin.

This study has demonstrated compelling results to motivate future work. One limitation is the relatively consistent body positioning of the participants (i.e., standing, working at a bench). While this is certainly a reasonable configuration for many occupational tasks, performance may suffer if the working posture changes significantly (e.g., working overhead). One strategy to improve model generalizability would be to generate synthetic inertial measurement units based on the optoelectronic motion capture data, as done by Mundt et al. for the lower limb [22]. This would permit for the augmentation of the training data by rotating the coordinates of the upper extremity relative to the global coordinate system. This is similar to the methods applied to image dataset augmentation (e.g., flipping, mirroring, shifting), however, the necessity of preserving the relationship between IMU local coordinate systems makes augmentation non-trivial. Using synthetic data would also allow the generation of examples in the underrepresented classes, which may improve the performance at high flexion angles.

The use of wearable sensors and machine learning for ergonomic assessment has a broad appeal, however, getting buy-in from practitioners requires substantive demonstration of utility. Here we have presented a method by which wrist joint postures can be estimated with an accuracy equal to that of a human practitioner. This approach has clear technical utility. It is our opinion that the most practical way to begin the integration of wearable technologies and machine learning in ergonomic assessment is to automate the tedious assessment tasks, which are currently performed by human practitioners. This will provide ergonomists and health and safety practitioners with more time to develop thoroughly informed interventions and exposure limits.

## 5. Conclusions

We have demonstrated a methodology for using convolutional neural networks to estimate wrist postures from inertial measurement units, and have shown that the method’s performance is congruent with human performance during occupational task wrist posture estimation. By predicting a granular component measure that practitioners may use in their analyses rather than attempting to estimate an overall level of risk, this method produces an output that is both practical to integrate within ergonomic analyses and comprehensible to practitioners. This also extends the functionality of IMU based measurement systems by providing a methodology for extracting angular measures, which does not rely on precise alignment or functional calibration movements.

## Figures and Tables

**Table 1:** Contingency table of cumulative model performance in flexion/extension with the true posture shown vertically and the predicted posture horizontally.

Flexion/Extension					
	θ>45°	45°≥θ>15°	15°≥θ>−15°	−15°≥θ>−45°	−45°≥θ
θ>45°	**0.30**	0.69		0.01	
45°≥θ>15°		**0.55**	0.39	0.03	0.03
15°≥θ>−15°		0.03	**0.64**	0.29	0.04
−15°≥θ>−45°			0.15	**0.65**	0.20
−45°≥θ			0.02	0.18	**0.80**

**Table 2:** Contingency table of cumulative model performance in radial/ulnar deviation with the true posture shown vertically and predicted posture horizontally.

Radial/Ulnar Deviation			
	θ≥10°	10°≥θ>−10°	−10°≥θ
θ≥10°	**0.58**	0.38	0.04
10°≥θ>−10°	0.14	**0.63**	0.23
−10°≥θ	0.04	0.43	**0.53**

## Data Availability

Access to data is restricted due to restrictions placed by the University of Guelph Research Ethics Board to protect the privacy of the study participants.

## Abbreviations

The following abbreviations are used in this manuscript:

CNN	Convolutional neural network
IMU	Inertial measurement unit
